# Mitochondria-Associated Endoplasmic Reticulum Membranes in Breast Cancer

**DOI:** 10.3389/fcell.2021.629669

**Published:** 2021-01-28

**Authors:** Hongjiao Yu, Chaonan Sun, Qing Gong, Du Feng

**Affiliations:** ^1^Department of Biochemistry and Molecular Biology, Guangzhou Medical University-Guangzhou Institutes of Biomedicine and Health (GMU-GIBH) Joint School of Life Sciences, Guangzhou Medical University, Guangzhou, China; ^2^Guangzhou Municipal and Guangdong Provincial Key Laboratory of Protein Modification and Degradation, State Key Laboratory of Respiratory Disease, School of Basic Medical Sciences, Guangzhou Medical University, Guangzhou, China

**Keywords:** mitochodnria, ER, MAM, breast cancer, carcinogenesis

## Abstract

Mitochondria-associated ER membranes (MAMs) represent a crucial intracellular signaling hub, that regulates various cellular events including Ca^2+^ homeostasis, lipid metabolism, mitochondrial function, and cellular survival and death. All of these MAM-mediated cellular events contribute to carcinogenesis. Indeed, altered functions of MAMs in several types of cancers have been documented, in particular for breast cancer. Over the past years, altered expression of many MAM-resident proteins have been reported in breast cancer. These MAM-resident proteins play an important role in regulation of breast cancer initiation and progression. In the current review, we discuss our current knowledge about the functions of MAMs, and address the underlying mechanisms through which MAM-resident proteins regulate breast cancer. A fuller understanding of the pathways through which MAMs regulate breast cancer, and identification of breast cancer-specific MAM-resident proteins may help to develop novel therapeutic strategies for breast cancer.

## Introduction

The interconnection between mitochondria and endoplasmic reticulum was the first inter-organelle contact site discovered in the late 1950's (Bernhard and Rouiller, 1956). Several subsequent fractionation studies have successfully isolated mitochondria-associated endoplasmic reticulum membranes (MAMs) as a distinct and purified structure from liver cells (Wanson et al., 1975; Pickett et al., 1981; Katz et al., 1983). These elegant studies provide the experimental evidence for the existence of MAMs. Both of endoplasmic reticulum (ER) and mitochondria play crucial roles in regulation of cellular homeostasis and cell death. The discovery of MAMs therefore leads to a growing research interest in characterization of the biological function of MAMs in cells. Over the past few decades, extensive researches have reported that MAMs act as signaling hubs that control ER and mitochondrial biology, and disturbances of MAMs result in dysfunctions in a wide range of cellular processes including apoptosis, inflammation, and autophagy (Morciano et al., 2018). These cellular activities have crucial roles in several pathologies including but not limited to cardiovascular disease and cancer. Recently, we have extensively reviewed the functional and mechanistic roles of MAMs in inflammation and cardiovascular disease (Liu et al., 2020). In the current review, we will highlight the multiple physiological functions of MAMs in mitochondrial Ca^2+^ signaling, metabolism and autophagy, and discuss how alterations in MAMs contribute to carcinogenesis.

## Structure and Biochemical Composition of MAMs

It is now widely accepted that MAM are 10–25 nm wide regions of juxtaposed membranes of ER and mitochondria tethered by proteins (Csordas et al., 2006), without complete fusion or loss of organelle identity. The biological functions of the MAMs are tightly regulated by the number, length and thickness of contacts between ER and mitochondria. The technique of biochemical purification of MAMs from different mammalian tissues and cultured cells has been greatly improved in the past years, and allows subsequent analysis of the composition of MAMs. The enrichment of several proteins including acyl-coenzyme A: cholesterol acyltransferase 1 (ACAT1/SOAT1), and the existence of Ca^2+^ nanodomains in MAMs have been identified (Rusinol et al., 1994; Vance, 2014). These data suggest that MAMs may have critical roles in regulation of lipid synthesis and Ca^2+^ trafficking. Since then, several studies have used high-throughput proteomics to identify novel proteins present in MAM purified from different cellular models (Poston et al., 2013; Cho et al., 2017; Hung et al., 2017). However, only two proteins, thioredoxin-related transmembrane protein (TMX1) and calnexin are commonly seen in the MAMs fraction from these studies (Simmen and Herrera-Cruz, 2018). It should be noted that not all proteins localized in the MAMs can be identified in the dynamic cellular environment. Nevertheless, a better characterization of the cell-specific MAM proteome with more novel techniques will definitely increase our current knowledge of the biochemical composition and biological functions of MAMs. Besides, Yang and colleagues presented a novel technique to detect MAM distribution and dynamics using split GFP protein as a reporter for labeling the ER membrane and mitochondrial outer membrane (Yang et al., 2018).

## Biological Functions of MAMs

In the past years, accumulating evidences have increased our knowledge of inter-organellar communication in the regulation of key cellular processes, such as metabolism and cell death. It is now widely recognized that the interaction between mitochondria and ER through the MAMs is critical in the various aspects of cellular health. In the next sections, we will discuss some of the vital cellular functions and biological processes regulated by MAMs.

## MAMs in Regulation of Ca^2+^ Signaling

**Figure 1:**
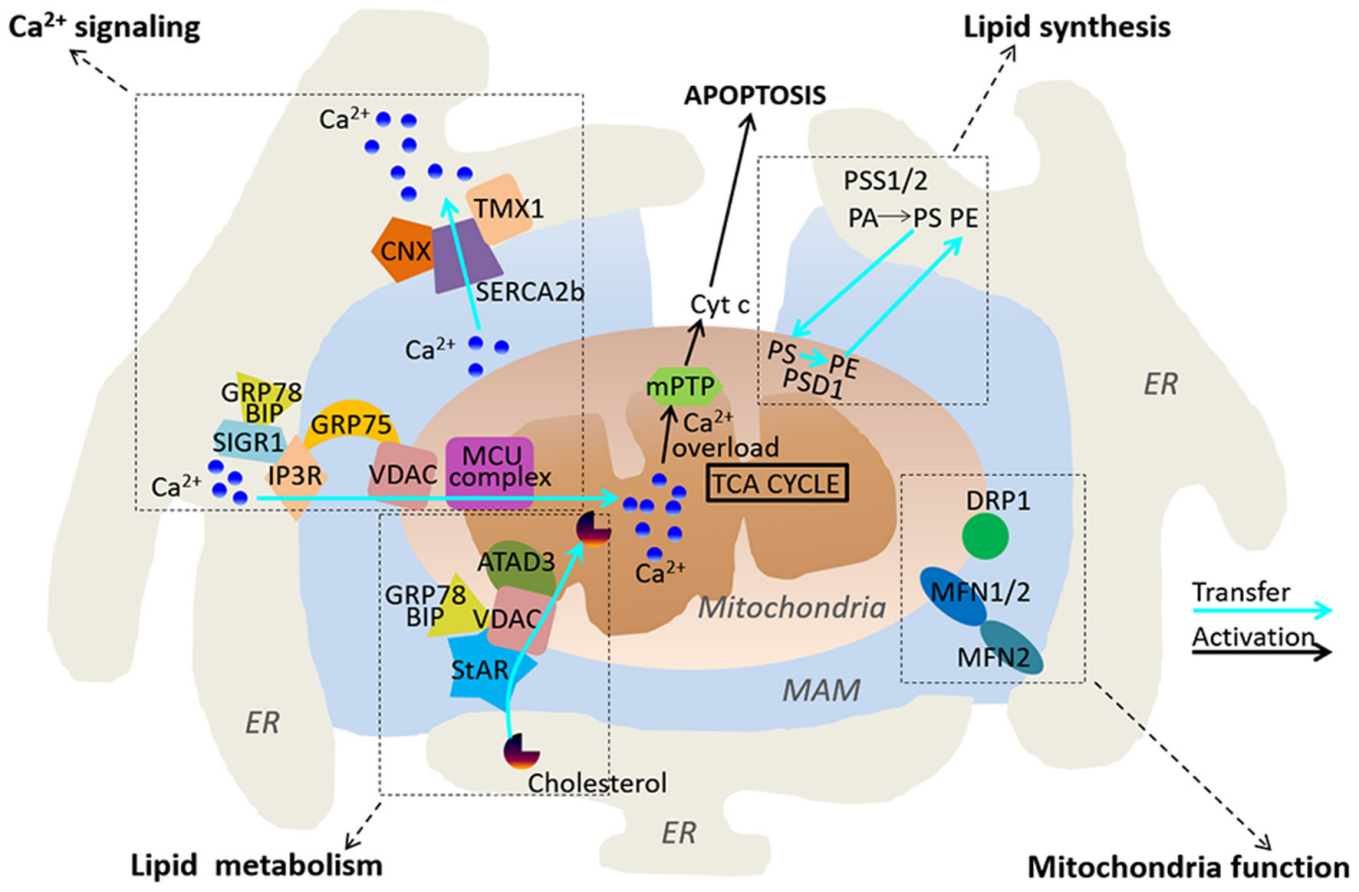
Functional roles of MAMs. MAMs play regulatory roles in Ca^2+^ signaling, lipid synthesis and metabolism, and mitochondria functions.

Ca^2+^ is an important intracellular second messenger, which is required for functional mitochondrial metabolism, bioenergetics and cell survival. MAMs have been shown to regulate a range of cell metabolism and cell fate determination through modulating cellular Ca^2+^ signaling (Figure 1, Rizzuto et al., 2009). ER is the main intracellular Ca^2+^ storage organelle in the cells. The tight tether between the ER membranes and mitochondria allows Ca^2+^ to be rapidly transferred between the two intracellular organelles (Rizzuto et al., 1998; Filippin et al., 2003). Indeed, the accumulation of Ca^2+^ in mitochondria is largely dependent on ER. Intriguingly, many ER associated Ca^2+^ handling proteins are enriched in MAMs, including inositol 1,4,5-trisphosphate receptors (IP3R) and the sarco/endoplasmic reticulum Ca^2+^ ATPase (SERCA). The IP3R together with the ryanodine receptors have been reported to be the principal ER Ca^2+^ channel (Parys and De Smedt, 2012). It appears that isoform 3 of the IP3Rs co-localizes most strongly with MAMs and acts as a primary regulator for transmitting Ca^2+^ signals to mitochondria (Mendes et al., 2005). It has been reported that the IP3Rs directly interact with the mitochondrial voltage-dependent anion channel 1 (VDAC1) located at the outer mitochondrial membrane (OMM) and associated to the MAMs (Szabadkai et al., 2006). The molecular chaperone glucose-regulated protein 75 (GRP75) regulates the interaction between VDAC1 and the IP3Rs, and it serves as a bridge which allows the transfer of Ca^2+^ from the ER to the Ca^2+^ permeable OMM. Unlike OMM, the inner mitochondrial membrane (IMM) is not permeable for Ca^2+^. Ca^2+^ located in the intermembrane space (IMS) is transferred to the mitochondrial matrix through the mitochondrial Ca^2+^ uniporter (MCU) (Baughman et al., 2011; De Stefani et al., 2011). The MCU complex is a multi-protein complex consisting of MCU, MCUb, EMRE, MICU1 and 2, MCUR1 and SLC25A3 (Mammucari et al., 2018). Each partner of the MCU multi-protein complex tightly controls Ca^2+^ concentration in the mitochondrial matrix, thereby regulating cell survival, death and metabolism.

The SERCA pump localized in the ER membrane regulates ER Ca^2+^ levels through actively pumping Ca^2+^ into the ER from cytosol, thereby leading to a Ca^2+^ gradient between cytosol and the ER. Interestingly, SERCA2b, an isoform of SERCA, exhibits the highest Calcium ions affinity, and is enriched in MAMs. It has been reported that calnexin and TMX1 regulate SERCA2b activity through a direct interaction with SERCA2b in a palmitoylation-dependent manner. Palmitoylation of calnexin switches its function from quality control of protein folding to ER-Ca^2+^ signaling by increasing the activity of SERCA2b, whereas TMX1 counteracts the interaction between Calnexin and SERCA2b, which inhibits SERCA2b and promotes Ca^2+^ influx to mitochondria (Gutierrez and Simmen, 2018). Another important protein present in MAMs is Mitofusin-2 (MFN2). It belongs to the GTPase protein family and plays a crucial role in regulation of mitochondrial fusion. MFN regulates the stability of ER-mitochondria interaction, Ca^2+^ and lipid transfer. It has been reported that loss of MFN2 reduces ER-mitochondria contact sites, and leads to impaired mitochondrial Ca^2+^ uptake (De Brito and Scorrano, 2008). However, there are discrepant results reporting that reduced or ablated MFN2 expression increases numbers of ER–mitochondria close contacts, and promote Ca^2+^ transfer between the two organelles (Filadi et al., 2015). The precise role of MFN2 in ER-mitochondria interaction therefore remains to be further investigated.

## MAMs Act as an Important Regulator of Lipid Synthesis and Metabolism

ER is the primary site for lipid synthesis. MAMs tightly regulate the lipid trafficking between ER and mitochondria (Figure 1). A number of proteins associated with lipid metabolism are enriched in MAMs, including phosphatidylserine synthases (PSS1 and 2), which is required for the synthesis of essential lipids such as phosphatidylserine (PS) and phosphatidylcholine (PC) (Futerman, 2006; Van Meer et al., 2008). The transfer of PS from ER to mitochondria via the MAMs is a key rate-limiting step in phosphatidylethanolamine (PE) synthesis. Previous studies have reported that reduced phosphatidylserine decarboxylase (PSD) activity causes accumulation of PS in MAMs (Voelker, 1984). These data indicate that MAMs play an important role in the transfer of PS between the ER and mitochondria. The long chain acyl-coA synthetase (ACSL4/FACL4) is a common marker used for characterization of MAMs. It regulates the ligation of fatty acids to coenzyme A and other cholesterol metabolites (Lewin et al., 2001). It has been previously reported that ACSL4 is localized in MAMs, and plays a key role in regulation of mitochondrial fusion during steroidogenesis in Leydig cells (Poderoso et al., 2013). In addition, cholesterol relative to the bulk of ER are enriched in MAMs and dysregulation of MAM cholesterol composition alters ER-mitochondria contacts (Fujimoto et al., 2012). Intriguingly, several enzymes that are associated with cholesterol metabolism and transport are found in MAMs, including ACAT1/SOAT1, and steroidogenic acute regulatory protein (StAR) (Rusinol et al., 1994; Prasad et al., 2015). Caveolin-1 (CAV1) is an important regulator of cholesterol intracellular transport and membrane organization, and it has been recently identified as a key component of MAMs. Genetic deletion of *Cav1* in mice reduces the stability of ER-mitochondria contact sites and an accumulation of free cholesterol in MAMs (Sala-Vila et al., 2016). Altogether, MAM appears to form a key molecular platform that plays an important role in regulation of lipid synthesis and metabolism.

## MAMs Regulates Mitochondria Function

Mitochondria morphology and dynamics are crucial in regulation of its diverse activity including mitochondrial respiration and metabolism, Ca^2+^ homeostasis and apoptosis. In response to various stimuli or stress, the morphology, mobility, and fusion-fission equilibrium of mitochondria are dramatically changed (Eisner et al., 2018). The underlying molecular mechanisms of mitochondrial fusion and fission have been extensively reviewed elsewhere (Wai and Langer, 2016), and will not be discussed in detail in this review.

As a microdomain between ER and mitochondria, MAMs not only regulate lipid synthesis and metabolism in ER, but also play a critical role in regulation of mitochondrial function. Indeed, several previous studies have revealed MAMs as a key regulator of mitochondrial dynamic and morphology (Figure 1). Dynamin-related protein 1 (DRP1) and MFN2 are well-established regulators of mitochondrial shape and fusion/fission balance. These two proteins have been found to be enriched in MAMs (De Brito and Scorrano, 2008; Saotome et al., 2008). As a most well-studied MAM-resident protein, MFN2 has been reported to regulate MAM structure and function. MFN2 locates at both OMM and the ER membranes, and it co-works with the OMM-associated MFN1 to establish homotypic and heterotypic ER–mitochondria interactions. Mitochondrial fission is induced at ER-mitochondria contact sites where ER tubules physically wrap around a part of the mitochondrial network and promotes mitochondria fission through the recruitment of DRP1 on the OMM (Friedman et al., 2011). The Miro family of proteins Miro1 and 2 are the master regulators of mitochondrial motility. They are located at the OMM and harbor two EF-hand Ca^2+^ binding domains, thereby sensing high levels of Ca^2+^ to regulate mitochondrial motility (Saotome et al., 2008). Miro1 and 2 have been shown to localize at the ER–mitochondria contact sites, where they sense local Ca^2+^ to connect the mitochondria to kinesin 1 (Fransson et al., 2006; Saotome et al., 2008).

The Ca^2+^ concentration in mitochondria plays a fundamental role in regulation of ATP production, which is required for a wide range of cellular processes. The Krebs Cycle, also known as the citric acid cycle, occurs within the mitochondria, which produces ATP through oxidative phosphorylation. The activities of key dehydrogenases of the Krebs Cycle are dependent on Ca^2+^, including the pyruvate dehydrogenase, the α-ketoglutarate, the isocitrate dehydrogenases and FAD-glycerol phosphate dehydrogenase (Hansford and Chappell, 1967; Rizzuto et al., 2004; Szabadkai and Duchen, 2008). The rapid exchange of Ca^2+^ in MAMs therefore has a critical role in regulation of mitochondrial bioenergetics, and subsequently cell fate determination (Cardenas et al., 2010). This view has been supported by several lines of evidences. Loss or gain-of-function studies have reported that MFN2, a key regulator of the ER–mitochondria contacts, significantly alters mitochondrial bioenergetics, which is independent of fusogenic function of MFN2 (Schrepfer and Scorrano, 2016). Deletion of MFN2 reduces several mitochondrial activities such as glucose oxidation, cellular respiration, mitochondrial membrane potential and proton leak, and mitochondrial coenzyme Q levels (Mourier et al., 2015), while overexpression of MFN2 increases mitochondrial metabolism (Lewis et al., 2016). Interestingly, MFN2 also transiently protects against starvation-induced autophagy and apoptosis through inducing mitochondrial hyperfusion and elongation (Lewis et al., 2016).

## MAMs and Cell Death

Although transportation of Ca^2+^ in MAMs benefits to mitochondrial bioenergetics, massive and prolonged mitochondrial Ca^2+^ overload can open the mitochondrial permeability transition pore (Szabadkai and Duchen, 2008). As a result, proapoptotic and caspase-activating factors in mitochondria are released in the cytoplasm such as including cytochrome c (Cyt c). Cytochrome c in the cytoplasm exacerbates Ca^2+^ release from IP3R by binding to it, thus avoiding the Ca^2+^-dependent inhibition of the receptor and amplifying caspase activation to promote apoptosis (Figure 1, Boehning et al., 2003). Further studies have demonstrated that tightening of the ER–mitochondria interaction increases mitochondrial Ca^2+^ uptake, leading to apoptotic cell death, while loosening of the ER–mitochondria interaction promotes mitochondrial respiration and cellular survival (Csordas et al., 2006; Marchi et al., 2014). MAMs may also induce apoptosis or ferroptosis through increasing reactive oxygen species (ROS), and subsequent the accumulation of lipid peroxidation products in cells (Verfaillie et al., 2012; Yang et al., 2014).

The proapoptotic BCL2 protein family members such as BAK and BAX mediate Ca^2+^ traffic from ER to mitochondria and exert a key role in regulating cell death (Scorrano et al., 2003). Over the past years an increasing amount of cancer-related proteins have been reported to be abundant in MAMs and to exert their antiapoptotic or proapoptotic functions through the manipulation of Ca^2+^ transfer and signaling between ER and mitochondria. IP3R, which plays a central role in the regulation of ER-mitochondria Ca^2^ transfer, is also a target under control of several oncogenes and tumor suppressors (Bittremieux et al., 2016). One of the main regulators of IP3R, the Akt serine threonine kinase, preferentially phosphorylates IP3Rs for its isoform 3 (IP3R3) in MAMs and blocks apoptosis by inhibition of IP3R-mediated ER-mitochondria Ca^2+^ release (Szado et al., 2008; Marchi et al., 2012). The antiapoptotic function of Akt is regulated by its inhibitors (PTEN and PML) and its mTORC2 activator, which are also enriched in MAM. The PTEN (phosphatase and tensin homolog) tumor suppressor is the dominant inhibitor of PI3K/AKT signaling pathways while the promyelocytic leukemia protein tumor suppressor (PML) functions in several apoptotic pathways. mTORC2/Akt at MAMs control apoptosis through interaction with the IP3R-Grp75–VDAC complex and regulation of IP3R3 phosphorylation and Ca^2+^ efflux from the ER to mitochondria (Betz et al., 2013).

The oncogenic transcription factor STAT3 has recently been determined enriched in ER and MAM. Constitutively active STAT3 induces cell resistance to apoptosis via reduced ER-mitochondria Ca^2+^ transfer and IP3R3 degradation mediated by IP3R3/STAT3 interaction (Avalle et al., 2019). The transcription factor p53 is one of the major tumor suppressors. It has been recently revealed that localization in ER and MAM enables p53 to mediate Ca^2+^ dependent apoptosis independent of its DNA-binding domain through interaction with SERCA. This interaction induces a change in SERCA oxidative state and facilitates Ca^2+^ uptake in the ER and ultimately impacts on sensitivity to apoptosis under stress conditions (Giorgi et al., 2015).

The long isoform of the cellular FLICE-inhibitory protein (c-FLIPL) has also been reported to localize at ER and MAM. As a well-known inhibitor of the extrinsic cell death initiator caspase-8, ablation of c-FLIPL disrupts ER-mitochondria juxtaposition, reducing ER-mitochondria Ca^2+^ transfer and affecting sensitivity to Ca^2+^ dependent apoptosis (Marini et al., 2015).

MAM appears as a crucial platform in autophagosome formation and plays an important role in autophagy. During starvation-induced autophagy, proteins involved in the autophagic machinery process such as ATG5 and ATG14, together with critical proteins involved in autophagosome formation such as Vps34 and Beclin 1, are recruited to MAM fraction (Hamasaki et al., 2013). Beclin 1 belongs to the class III phosphatidylinositol 3 kinase complex (PI3K complex III) and is essential for autophagy induction as well as autophagosome formation. Function of Beclin 1 is tightly regulated through direct binding of autophagy inducing or inhibiting regulators, such as autophagy/Beclin 1 regulator 1 (AMBRA1) and BCL2 (Kang et al., 2011). Interestingly, Ca^2+^ signaling appears to act as an activator and meanwhile as an inhibitor of autophagy (Bootman et al., 2018). IP3R is a key negative regulator of autophagy which is directly targeted by Beclin 1 and promotes the formation of a complex with Beclin 1/BCL2. The pool of soluble Beclin 1 protein for autophagy induction is therefore decreased by the BCL2-mediated sequestration of Beclin 1 (Vicencio et al., 2009). However, further studies demonstrated that IP3R enhances autophagic pathway through formation of IP3R/Beclin 1 complex. After starvation, increased Beclin 1 interacts with IP3R and promotes IP3R-mediated Ca^2+^ signaling by enhanced ER-Ca^2+^ stores content through upregulation of GRP78/BiP and calreticulin (Decuypere et al., 2011).

MAM also plays a critical role in inflammation pathways through recruitment of the NOD-, LRR- and pyrin domain-containing protein 3 (NLRP3) to the MAM faction from cytosol. Stimulus including extracellular ATP and impaired Ca^2+^ flux from ER to mitochondria trigger the production of ROS and ultimately activate the NLRP3 inflammasome. Activated NLRP3 inflammasome recruits apoptosis-associated speck-like protein containing a CARD (ASC) and relocates to the MAM fraction, which finally results in caspase 1-dependent release of proinflammatory cytokines IL-1β and IL-18 and leads to Gasdermin D-driven pyroptotic cell death (Tschopp, 2011). Details on NLPR3 activation and regulation have been excellently reviewed elsewhere and will not be discussed here (Missiroli et al., 2018; Swanson et al., 2019).

## Functional Alterations of MAMs and its Regulatory Role in Breast Cancer

As discussed above, given the fact that MAMs play critical roles in regulation of cell homeostasis, survival and apoptosis, it is not surprising that MAMs are functionally altered during carcinogenesis. Indeed, several tumor suppressors or oncogenes have been found to target MAMs to regulate carcinogenesis. Here, we discuss the emerging evidence showing the critical roles of MAMs in breast cancer development.

**Figure 2:**
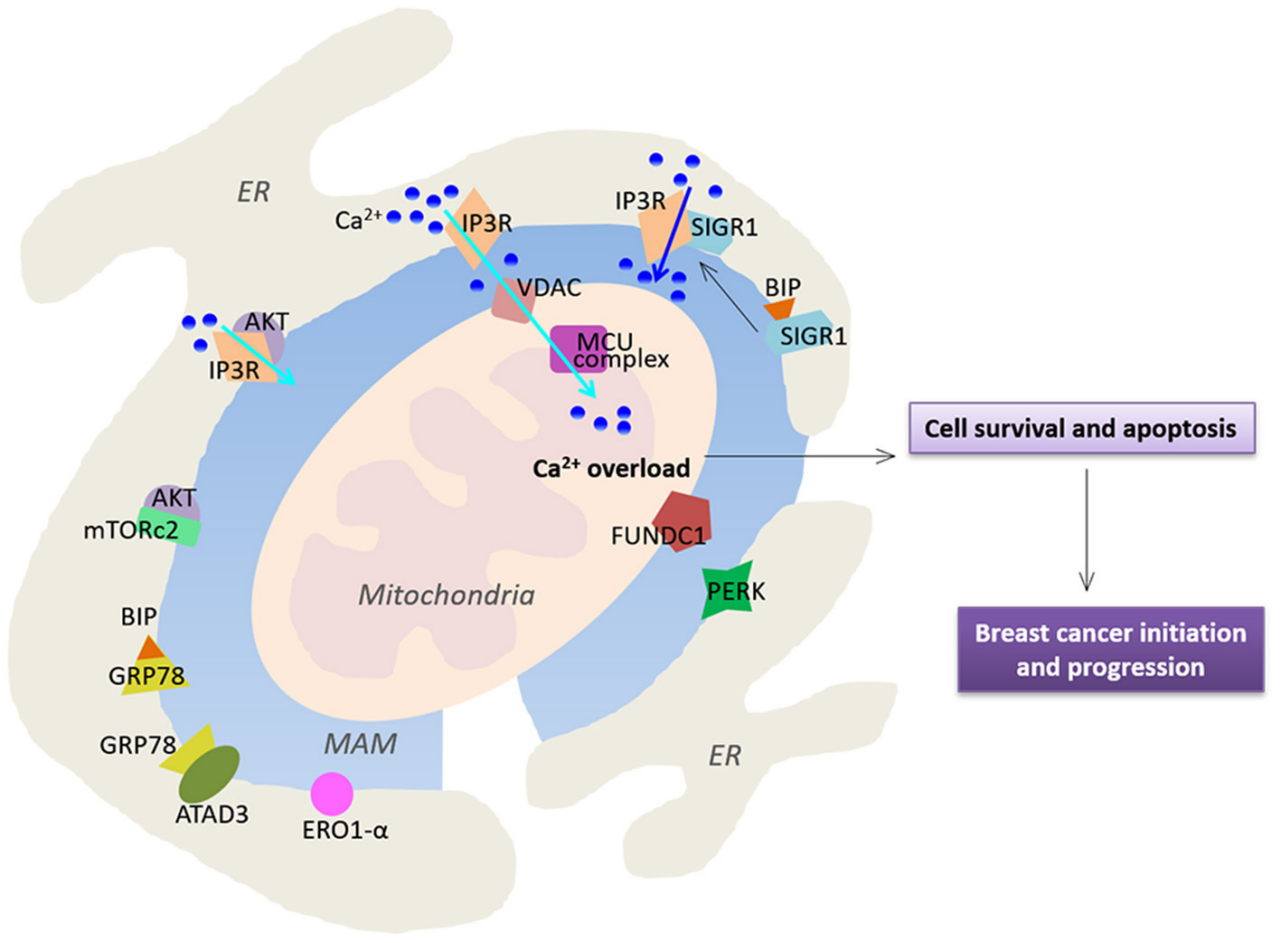
Functional alterations of MAMs in breast cancer. MAMs-resident proteins play a crucial role in regulation of Ca^2+^ homeostasis, thereby affecting cell survival and apoptosis. Cyan arrows highlight Ca^2+^ transport while the blue arrow indicates a larger Ca^2+^ fluxes. See text for further details.

Breast cancer is one of the most common cancers in the world. MAMs have critical roles in the onset of breast cancer, which reprogram normal cell signaling toward malignancy. Abnormal expression and localization of MAM-resident proteins have been widely reported in breast cancer (Figure 2). For example, the stress-activated chaperone sigma-1 receptor (Sig1R) at the ER-mitochondria interface has been reported to be expressed higher in metastatic potential cancer cells compared to that in normal tissues (Aydar et al., 2006; Gueguinou et al., 2017). It has been previously reported that Sig1R binds the MAM chaperone BiP/GRP78 under physiological conditions. However, Sig1R dissociates from BiP and binds IP3R3 upon activation of IP3Rs, which stabilize IP3R3 at the MAMs and enhance IP3R3-mediated Ca^2+^ fluxes to the mitochondria to regulate cell survival (Hayashi and Su, 2007). Importantly, in response to chronic ER stress involving prolonged ER Ca^2+^ depletion, Sig1R can translocate from MAMs to the peripheral ER, causing reduced cell damage and death (Hayashi and Su, 2003, 2007). In addition, Sig1R also involves in cell invasiveness in breast cancer. Sig1R can form a functional molecular platform with the calcium-activated K+ channel SK3 and Orai1, which drives Ca^2+^ influx to promote the migration of cancer cells (Gueguinou et al., 2017).

As mentioned before, MAMs have critical roles in regulation of Ca^2+^ signaling in cells. Indeed, Ca^2+^ fluxes toward the mitochondria via MCU are crucial for tumor growth and metastatic behavior. It has been previously reported that silencing of MCU inhibits cell migration and invasion. In triple-negative breast cancer, MCU prevents tumor progression in MDA-MB-231 xenografts and regulates metastasis via a key regulator hypoxia-inducible factor 1 (HIF1) which controls gene reprogramming (Tosatto et al., 2016)

Furthermore, IP3R3 is highly enriched in MAMs, and is recognized as a MAM marker (Wieckowski et al., 2009). IP3R3 transmits Ca^2+^-mediated proapoptotic signals to the mitochondria, and also plays an important role in regulation of cellular bioenergetics and metabolism in breast cancer (Mendes et al., 2005). Inhibition of IP3R3 expression leads to autophagic breast cancer cell death, and mitotic catastrophe in tumorigenic cells but not in normal cells (Bultynck, 2016; Cardenas et al., 2016; Singh et al., 2017a). Corroborating with these observations, higher expression of IP3R3 in human malignant tissues and higher concentrations of metabolites in serum samples have been reported in breast cancer patients (Singh et al., 2017b). On the other hand, IP3R3 function is also regulated by a wider range of oncogenes and tumor suppressors such as the oncogene Akt kinase (Khan et al., 2006; Szado et al., 2008). Altered PI3K/Akt/mTOR pathway is frequently seen in human breast cancers (Stemke-Hale et al., 2008; Gonzalez-Angulo et al., 2011). Selective phosphorylation of IP3R3 by AKT reduces ER-mitochondria Ca^2+^ transfer and attenuates apoptotic responses (Marchi et al., 2012). The mechanistic TOR complex 2 (mTORc2) is located in MAMs, where it phosphorylates Akt at position S473 (Sarbassov et al., 2005; Betz et al., 2013). The essential role of mTORc2-Akt signaling in maintaining proper MAM functionality has been demonstrated. Loss of mTORc2 induces disruption of MAM architecture and mitochondrial defects (Betz et al., 2013). Consistently, it has been reported that expression of the mTORc2 core component Rictor is significantly up-regulated in invasive breast cancer specimens, which promotes Akt-dependent tumor progression in HER2-amplified breast cancers (Morrison Joly et al., 2016).

The role of MAM chaperone BiP/GRP78 in the development of breast cancer has also been reported. ATAD3a is a mitochondria and MAM resident protein with unknown function. GRP78 has been reported to cooperate with ATAD3a to stabilizing WASF3, a protein that facilitates actin polymerization, thereby enhancing invasion and metastasis (Teng et al., 2016). ATAD3a regulates ER-mitochondria contact site formation and cholesterol substrate delivery to the mitochondria (Issop et al., 2015). Importantly, dysregulation in lipid composition of MAMs results in severe consequences, affecting Ca^2+^ homeostasis, ER-mitochondria contact sites, and mitochondrial functions (Vance, 2014), which are known to regulate cell apoptosis and tumors. Importantly, a number of previous studies have reported that enhanced lipogenesis in cancer cells promotes mass growth (Zaidi et al., 2013).

Endoplasmic reticulum oxidoreductin 1-α (ERO1-α) is an oxidizing enzyme that is enriched in MAMs (Anelli et al., 2012). ERO1-α controls oxidative folding and ER redox homeostasis, and regulates ER Ca^2+^ flux and subsequent mitochondrial Ca^2+^ accumulation, which has been reported to be highly expressed in several types of cancer (Kakihana et al., 2012). Notably, ERO1-α has been reported to be a novel predictor for poor prognosis of breast cancer (Kutomi et al., 2013). ERO1-α mediated ER-mitochondria Ca^2+^ flux activates the procaspase activating compound-1 (PAC-1) to induce ER stress and mitochondrial permeabilization, thereby promoting apoptosis in a variety of cancer cell types (Seervi et al., 2013). Interestingly, it has been reported that ERO1-α expression is correlated with programmed cell death ligand 1 (PD-L1) in triple-negative breast cancer cells. Ablation of ERO1-α causes significantly reduced PD-L1–mediated T-cell apoptosis, indicating ERO1-α may be involved in tumor-mediated immunosuppression (Tanaka et al., 2017).

RNA-dependent protein kinase (PKR)–like ER kinase (PERK), a critical ER stress sensor at MAMs, has been shown to play a key role in maintaining the ER-mitochondria juxtaposition and ROS-mediated mitochondrial apoptosis (Verfaillie et al., 2012; Van Vliet et al., 2017). PERK-dependent signaling involves in tumor initiation and expansion to preserve redox homeostasis and promote tumor growth in MDA-MB-468 and T47D cell lines (Bobrovnikova-Marjon et al., 2010). Silencing of PERK expression reduces tumor growth and restores sensitivity to chemotherapy in resistant tumor xenografts (Salaroglio et al., 2017).

FUN14 domain-containing protein 1 (FUNDC1) is a novel outer mitochondrial membrane protein localized in MAMs (Wu et al., 2016, 2017). It regulates the formation of MAMs and elicits Ca^2+^ release from ER into mitochondria and cytosol in mouse cardiomyocytes (Wu et al., 2017). Several studies have shown that overexpression of FUNDC1 induces mitophagy in various cancer cell lines (Hirota et al., 2015; Hui et al., 2019; Roperto et al., 2019). Interestingly, a recent study has reported that FUNDC1 enhances breast cancer proliferation and migration via Ca^2+^ influx-mediated NFATC1 activation and translocation into the nucleus to activate BMI1 transcription (Wu et al., 2019). Notably, FUNDC1 expression is positively associated with worse disease progression in breast cancer (Wu et al., 2019).

## Conclusion and Future Perspectives

In conclusion, MAMs are critical regulators of intracellular signaling pathways and regulate various biological functions such as Ca^2+^ homeostasis, lipid metabolism, mitochondrial function, and cell death. Over the past years, targeting of cell death pathways is a commonly used therapeutic strategy in the treatment of breast cancer. Cell-death-pathway-involved proteins in MAMs such as BCL2, p53 tumor suppressor, NLRP3 and their related proteins have been widely investigated for prognosis and targeted anti-cancer therapy. With increasing evidences support a critical role of MAMs in the tumorigenesis of breast cancer, understanding the mechanisms and functions of the mitochondria-ER interface are particularly important and urgent. It is likely that there might be different ER-mitochondria contact sites in different types of cells, which leaves many unanswered questions. One crucial question is that whether there are properties of MAM-resident proteins that are uniquely associated with one specific type of cancer.

Functional MAM alterations appear to represent a new therapeutic strategy to target cancer cells by suppressing their survival and invasion, however, it will be a major challenge to limit these effects to cancer cells without affecting normal cells, identification of potential breast cancer-specific MAM proteins is therefore of decisive importance for the accurate therapeutic target in cancer treatment. Specific targeting of cancer-altered MAM function, dynamics or structures also open new perspectives toward other types of human metabolic disorders that involve MAM pathways.

## Author Contributions

As the first authors, HY and CS make the same important contributions to design concept, collected and analyzed materials, wrote articles and drew. DF and QG make the final review and finalization of the articles to be published as the corresponding authors. All authors contributed to the article and approved the submitted version.

## Conflict of Interest

The authors declare that the research was conducted in the absence of any commercial or financial relationships that could be construed as a potential conflict of interest.
